# Concept Maps for Teaching, Training, Testing and Thinking

**DOI:** 10.15694/mep.2020.000171.1

**Published:** 2020-08-19

**Authors:** Prashanti Eachempati, Komattil Ramnarayan, Kiran Kumar KS, Anoop Mayya

**Affiliations:** 1 Melaka Manipal Medical College; 2 Melaka Manipal Medical College

**Keywords:** Concept maps, Teaching and learning strategies, Critical thinking, Assessment, Training, Meaningful learning

## Abstract

This article was migrated. The article was marked as recommended.

Concept maps are evidence based pedagogical tools to fathom how meaningfully students have accomplished their learning objectives. They also give intuitive insights to improvise instruction to enable better and deeper understanding the foundations of learning. In this paper we provide an overview of concept maps and share our experiences of using concept maps for the 4 t’s of education - teaching, training, testing and thinking.

## Introduction

The cognitive processes undergirding teaching- learning are so distinctly dissimilar that it is important to use variegated tools to enhance knowledge construction in the learners. Pedagogical changes have been propounded, with an aim to transform the student into a critical, reflective individual who, in his practice, is able to ‘fully learn how to learn’ ( Gomes *et al*., 2011). This article on concept maps explores one such pedagogical intervention.

As defined by Torre, Durning and Daley (2013) “ *Concept mapping is an instructional strategy for individual and group learning that involves integration of knowledge and creation of meaning by relating concepts.*” Moreover, concept maps help learners to organize and represent ideas so that they can reflect on their learning, leading to a deeper understanding. Concept maps can be used not just for teaching and testing but also for training and thinking - all of which represent a learning continuum.

## Initiation of Concept Maps

**Figure 1.**
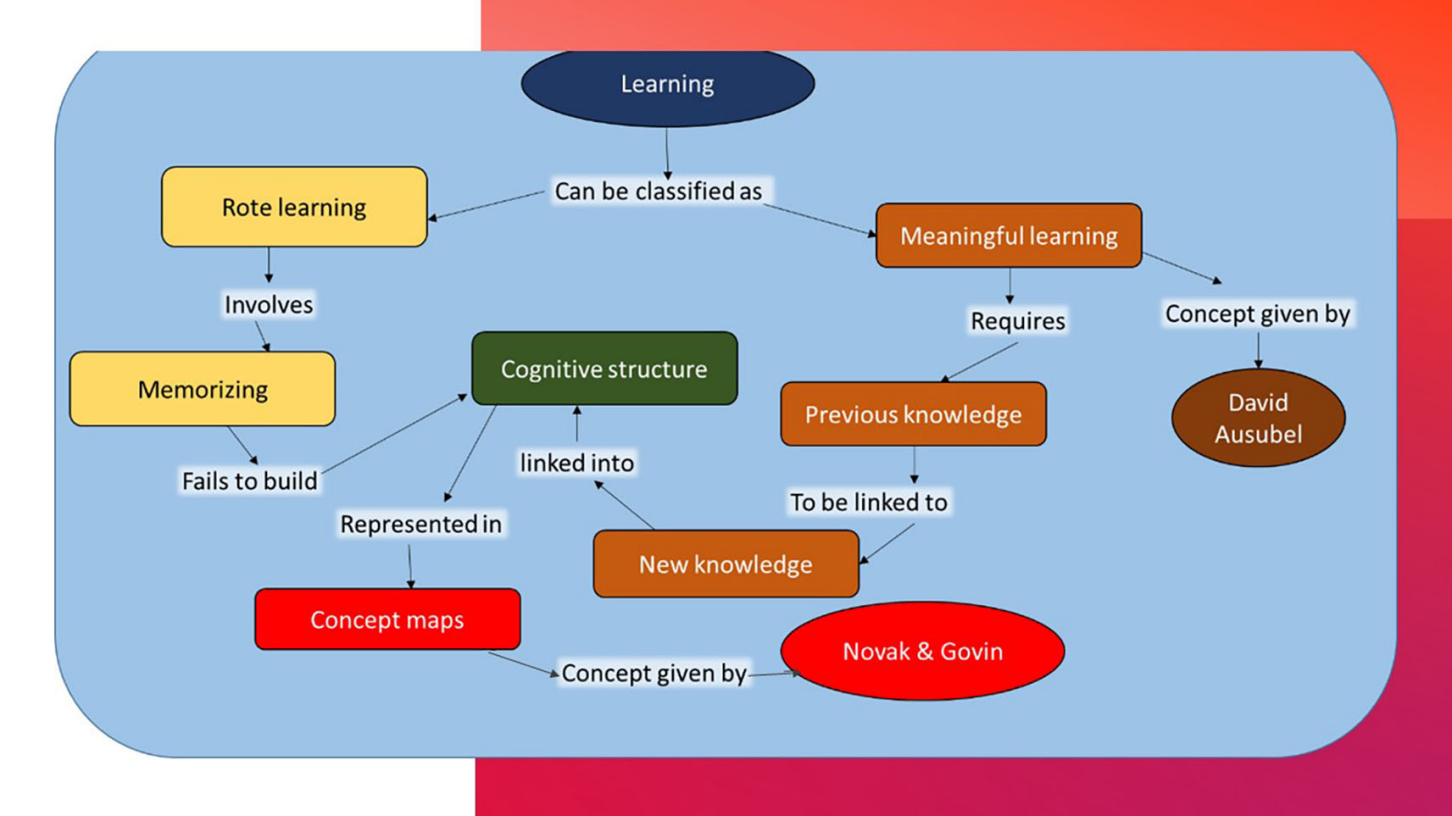
Ausubel’s theory represented as a Concept Map

Novak and Canas (2006) and collaborators developed concept maps from David Ausubel’s theory called the ‘meaningful learning theory’ which proposed that knowledge construction is based on relevance and integration and not on arbitrariness or rote learning ( Figure 1).

**Figure 2.**
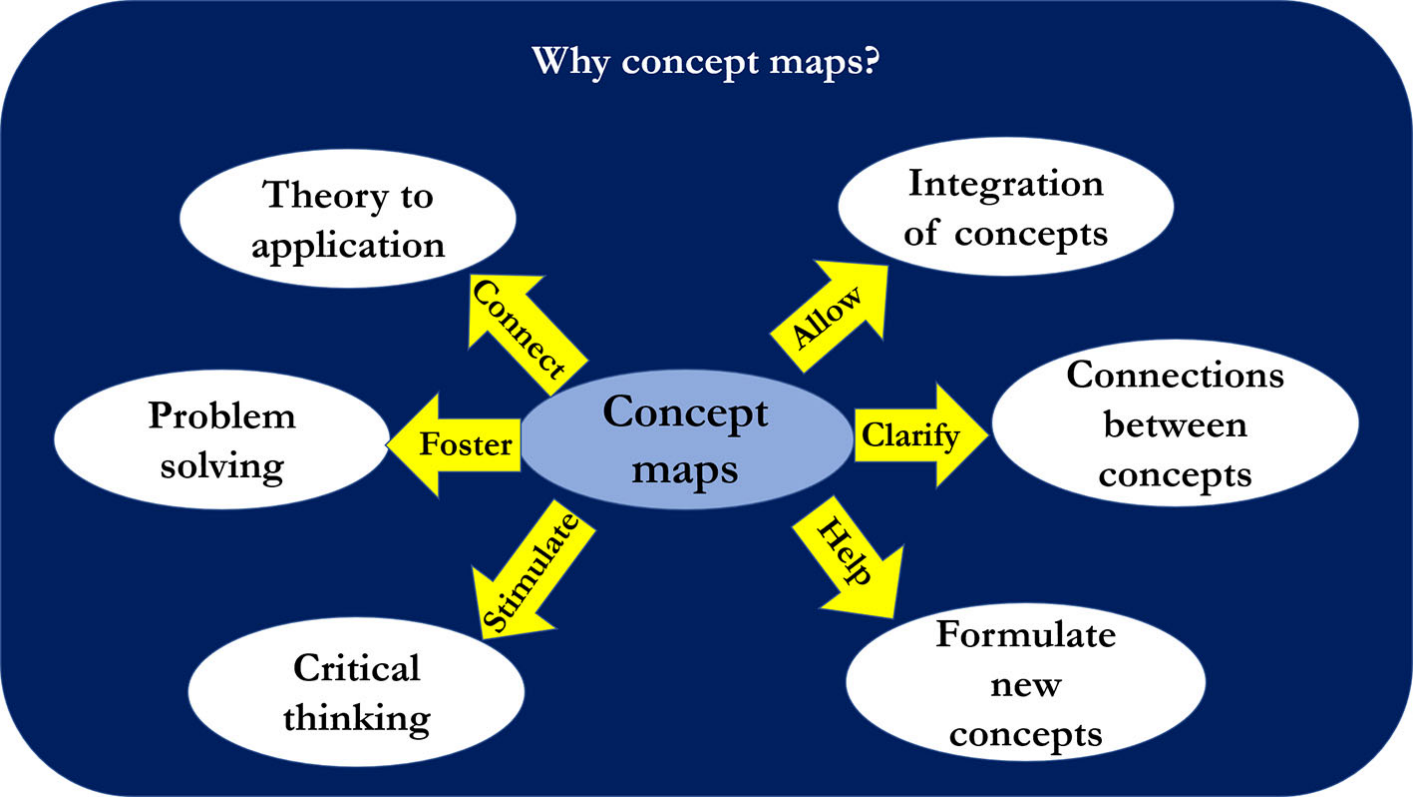
Uses of Concept Maps

Ausubel reasoned that pre-existing cognitive structures promote assimilation of the new proposals and nurtures connection between concepts, allows development of new concepts and integration of concepts ( Gomes *et al*., 2011). Concept maps when used appropriately promote critical thinking and problem-solving capabilities allowing students to convert theoretical into application-based knowledge ( Figure 2).

## How to construct a Concept Map

**Figure 3.**
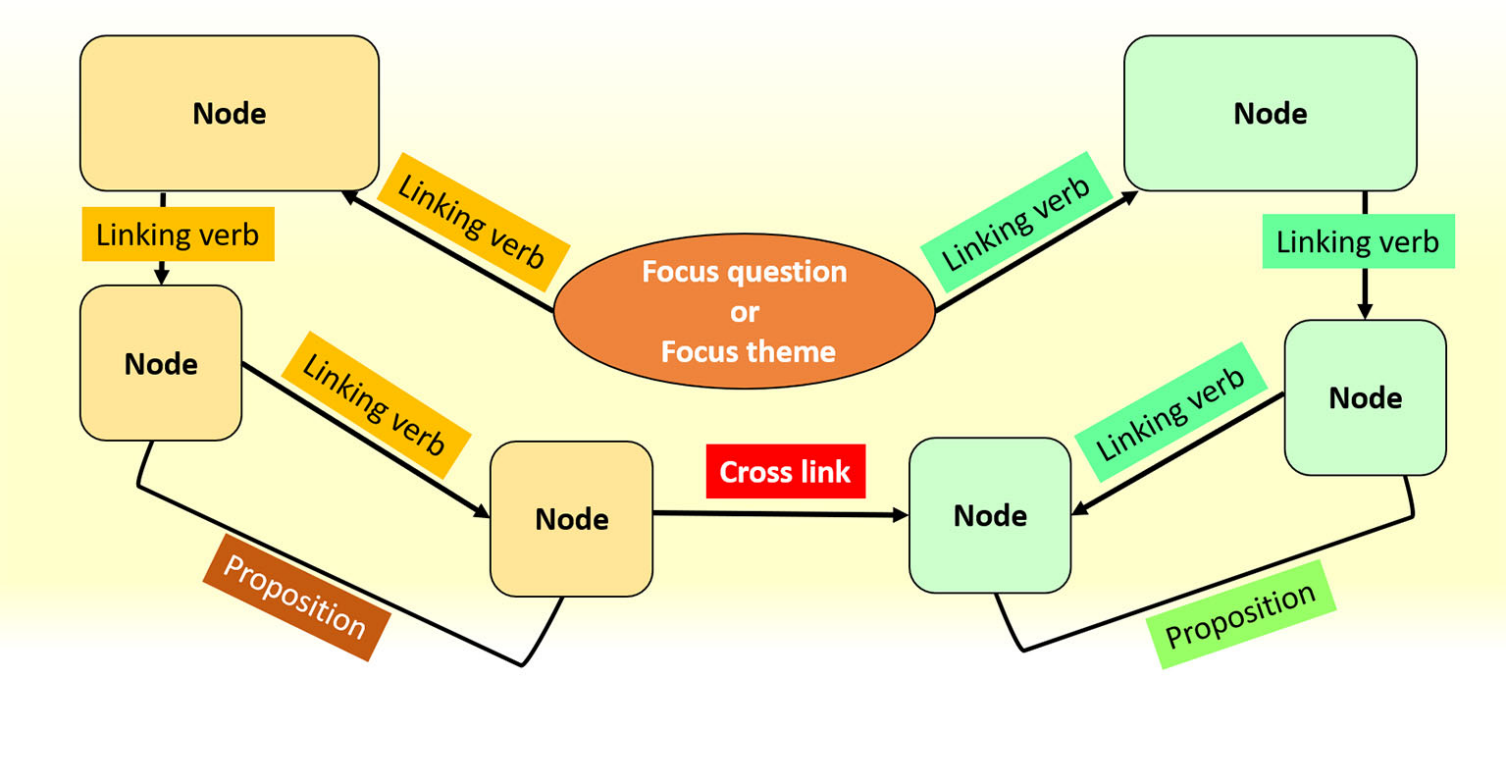
Structure of Concept Maps

The structure of a concept map is made up of nodes, linking verbs, cross-links and propositions. The complete structure of a concept map is built around a focus question or theme. Every concept or idea is represented as a word or short phrase and lies inside a box which is called a *node.* A *linking verb* connects the nodes and explains the relationship between them. *Cross-links* are relationships between concepts in different domains of the concept map, allowing us to visualize the connection between them. *Proposition* of a concept map involves two nodes and their linking verbs; a proposition should form a meaningful sentence and represents the smallest unit in the map ( Gul and Boman, 2006; Gomes *et al.*, 2011) ( Figure 3).

Prior to the drawing of a concept map, it will be worthwhile to generate a list of the key concepts that need to be included. This list must be in a rank order from the most general concept to the most specific. This list is referred to as a *parking lot*, as items are picked from here and moved into the map accordingly. The interrelationship between concepts is critical. This requires meticulous usage of cross-links and precision in the choice of linking words.

## Types of Concept Maps

Organization of concept maps depends on the creativity and innovation of the individual drawing them. Different types of concept maps have been described in the literature ( Gomes *et al*., 2011).

**Figure 4.**
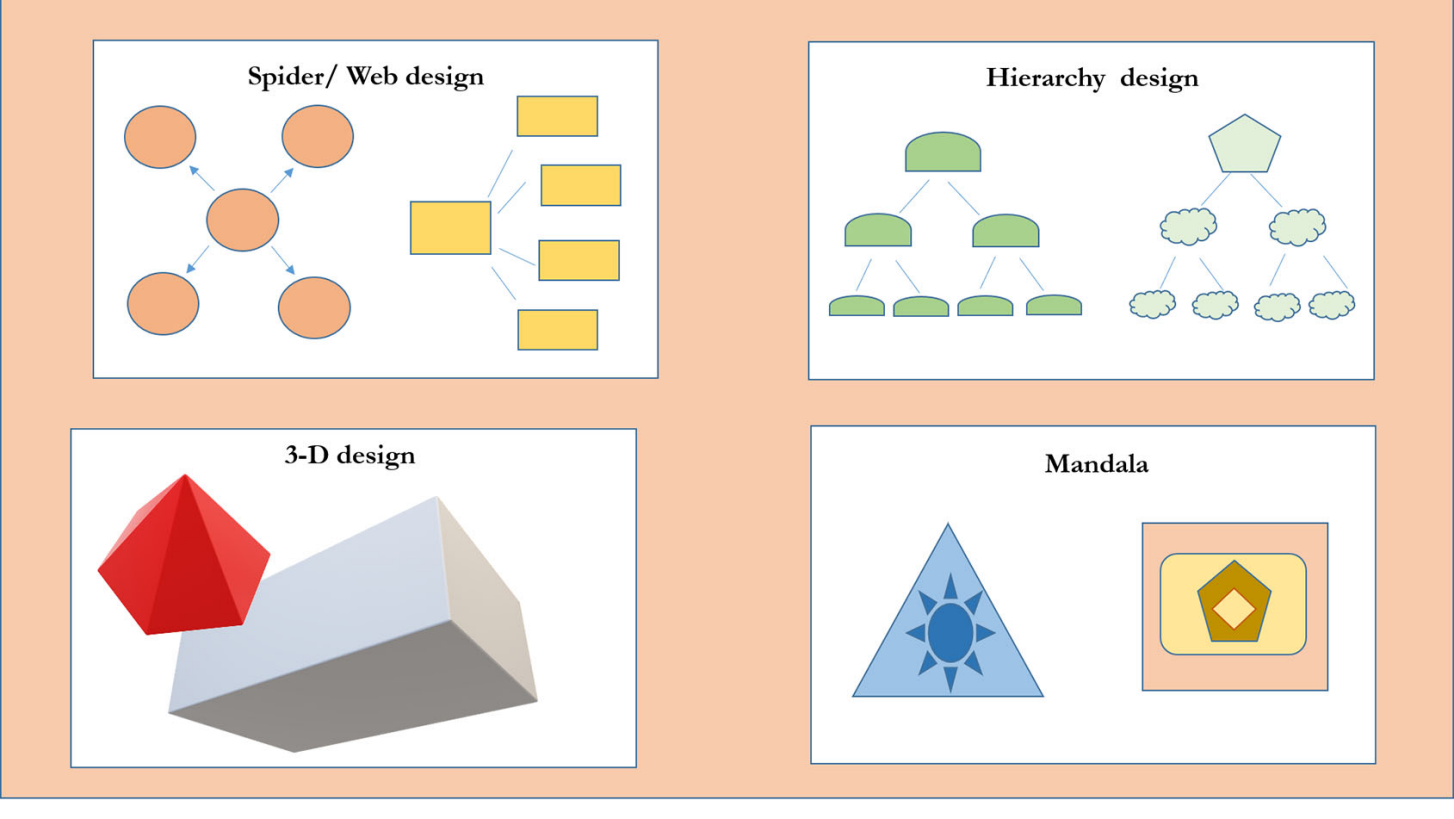
Examples of different types of Concept Maps

• **Spider/ Web design:** in which the center represents the focus question or theme with linking words spreading out to connect different nodes depicting a spider web.
• **Hierarchical design:** where the information is represented as a scale of importance usually in the descending order.
• **Flowchart:** where the information is represented in a linear fashion.
• **Conceptual way:** which is very similar to a flowchart, with the possibility of adding new concepts or deleting some.
• **Landscape structure:** which displays information in panoramic manner.
• **Multidirectional structure (3-D):** which uses depth to represent relationships that cannot be shown with 2-dimensional maps.
• **Mandala:** in which information is presented in geometric forms ( Figure 4).

It is vital to understand that concept maps are not rigid, although they are a product of logical thought. The maps are very flexible and are in constant change as new knowledge is acquired ( Gul and Boman, 2006; Gomes *et al*., 2011).

### Digital Concept Maps

Digital concept maps facilitate real time interaction and feedback between learner and instructor. The digital platform allows learners to include images, photos and hyperlinks (to other websites) to support the content ( Chang, Sung and Chen, 2008). It also provides the flexibility to co-create and co-edit concepts more easily ( Gijlers and Jong, 2013). For today’s student generation of technophiles these applications are akin to putty in their hands for creating concept maps thus enabling more focus on knowledge construction than on its designing ( Chiu, Huang and Chang, 2000). Of the numerous online concept map applications available, Mindmup, Gliffy, Mindmeister, Bubbl and Minddomo are some of the commonly used ones ( Chiu, Huang and Chang, 2000; Gijlers and Jong, 2013).

## Blueprinting for constructing Concept Maps

**Figure 5.**
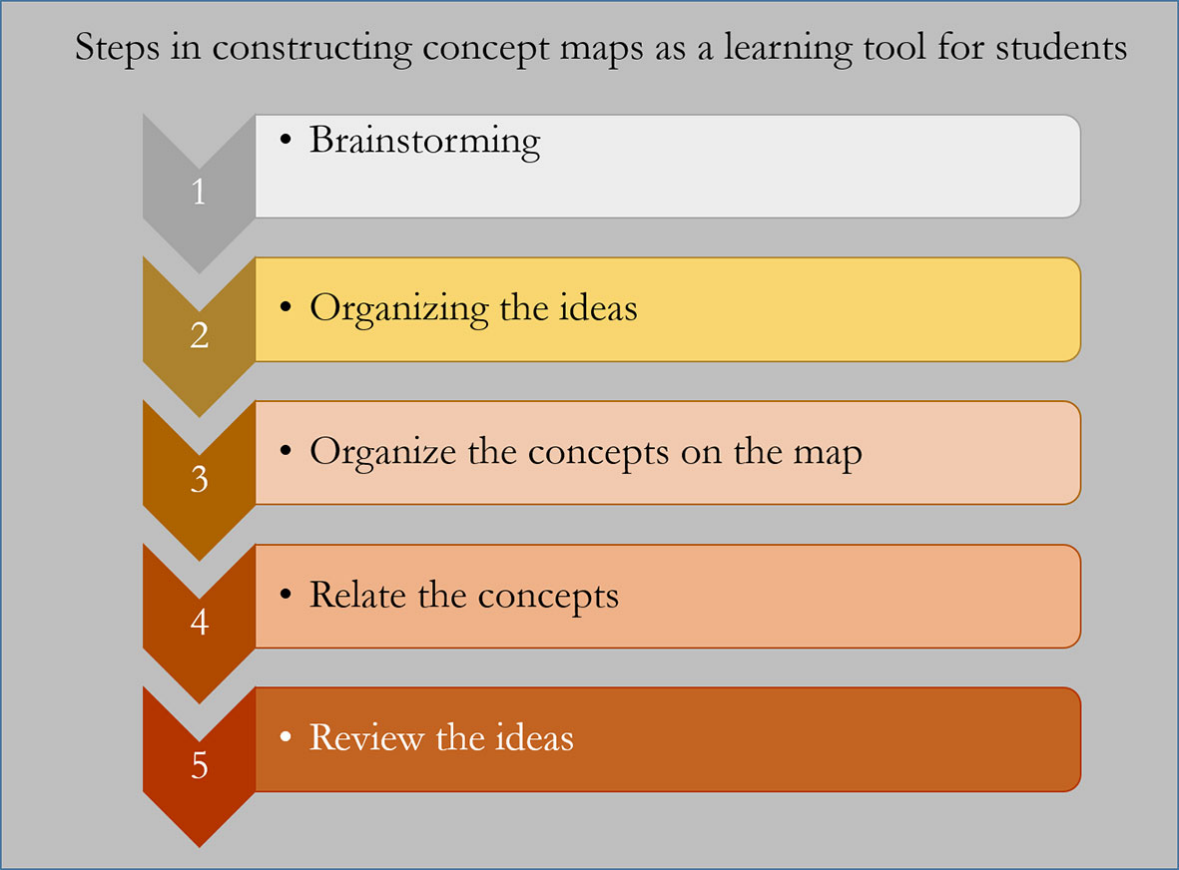
Blueprint for constructing Concept Maps for students

The purpose, the conceptual clarity and prior planning will determine how concept maps are designed. When concept maps are used for learning, the primary step is a list building exercise through brainstorming. Following this, the items in the list can be grouped and sub-grouped. The key concepts can be generated from these groups. Attempts can now be made to link and cross-link. Finally, the concept map can be reviewed to ascertain if it faithfully reflects the thought process ( Croasdell, Freeman and Urbaczewski, 2003) ( Figure 5).

**Figure 6.**
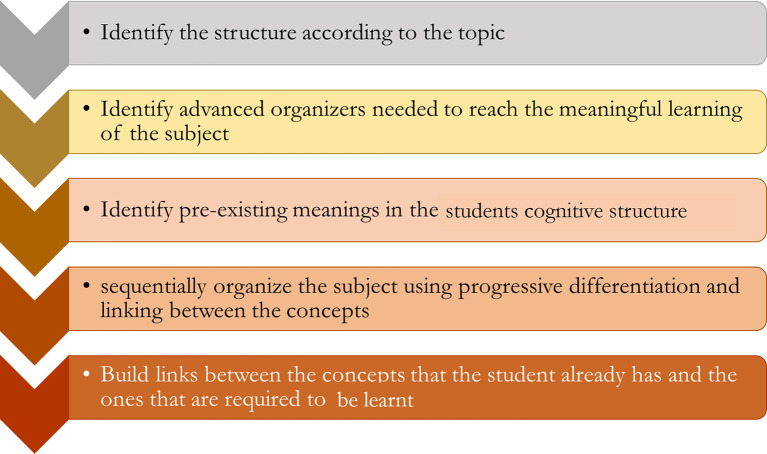
Blueprint for Concept Map construction for teachers

When a teacher used maps to conceptualize, it is also an opportunity to involve students in meaningful linking of the concepts ( Croasdell, Freeman and Urbaczewski, 2003). From the teacher’s viewpoint, there is a stepwise approach to generating concept maps ( Figure 6).

## Our experience with Concept Maps

We used concept maps in the departments of Prosthodontics and Pathology for teaching, training, testing and thinking. The ethical approval was obtained from Institutional Research Ethics Committee -Ref No: MMMC/FOD/AR/EC 2018 (F-03). Consent to use the images was obtained from the participants.

### Concept Maps for teaching

The literature abounds in evidence supporting concept mapping as a meta-cognitive strategy that enhances meaningful learning ( Novak, 1990; Irvine, 1995; Pintoi and Zeitz, 1997).

**Figure 7.**
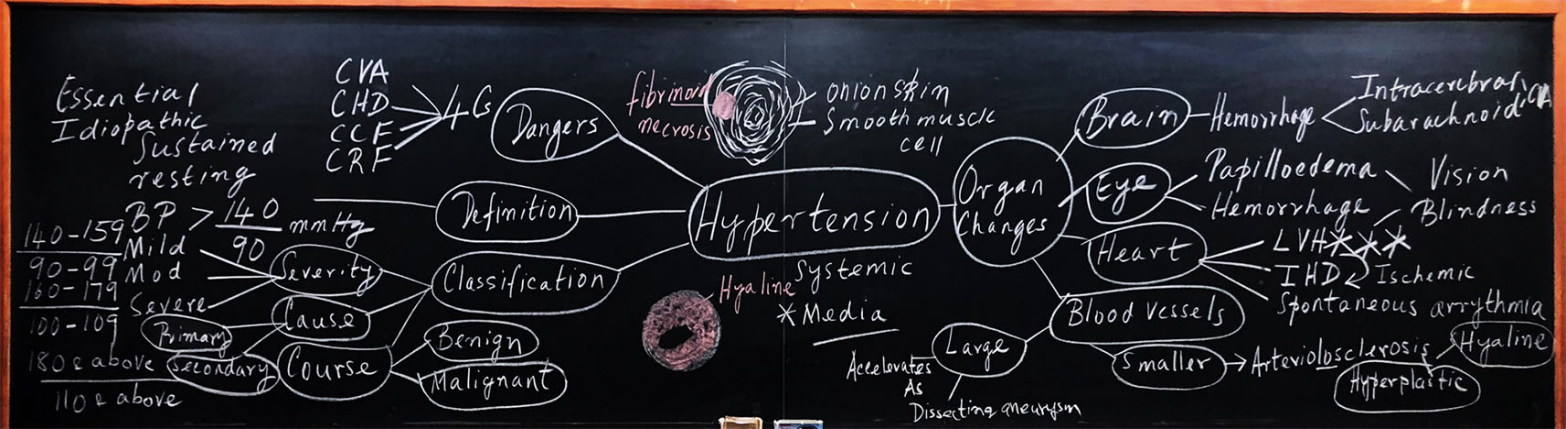
Concept Map on hypertension used as a teaching tool

While using concept maps, conceptual reinforcement takes precedence over content coverage as students learn to establish meaningful relationships between individual concepts. What emanates is a type of ‘inferential and analogical reasoning’ so essential for success ( Mintzes, Wandersee and Novak, 2007). The aforesaid is exemplified in a pathology class, where it was used as a pedagogical tool to provide a panoramic sketching of hypertension ( Figure 7).

We also tried an experimental study where students were divided into two groups and were taught using traditional and concept map approaches. At the end of the teaching module, students in both the groups were tested using multiple-choice questions (MCQs) comprising recall and reasoning level questions. The performance of the students was the same in both groups on the recall level MCQs but the concept map approach group outperformed the traditional approach group in the reasoning level MCQs (Results in Table 1).

**Table 1.** Comparison of test scores

MCQ Type	Method	n	Mean SD	P -Value *
Reasoning	Traditional	68	6.13±0.98	<0.001
	Concept Map	68	7.00±1.22	

* unpaired t-test

These results were immediately after the teaching session and we are aware that the novelty of the approach could have influenced the results. Our hope is that this method will provide the sensitization and scope to train the students to constantly make links between concepts, thus enhancing their critical thinking.

### Concept Maps for training

Training differs from teaching in that the emphasis is on development of abilities rather than on conveying information or sharing knowledge ( Surbhi, 2019). Training students to represent their thinking in their concept maps is vital. We used concept maps not only for teaching but also to train students in becoming adept at drawing the maps independently.

All and Huycke, (2007) suggested the use of serial maps for training students. Serial maps are concept maps drawn by students at different stages during the learning, thereby allowing teachers to monitor their students’ progress through constructive and timely feedback.

We used different techniques to train students to draw concept maps. As the students were already exposed to concept map teaching, they were aware of the processes involved in drawing the map. They were encouraged not only to prepare notes but also to take down notes in the classroom in the form of concept maps. In other words, they were encouraged to use concept maps in both note-making and in note-taking.

**Figure 8.**
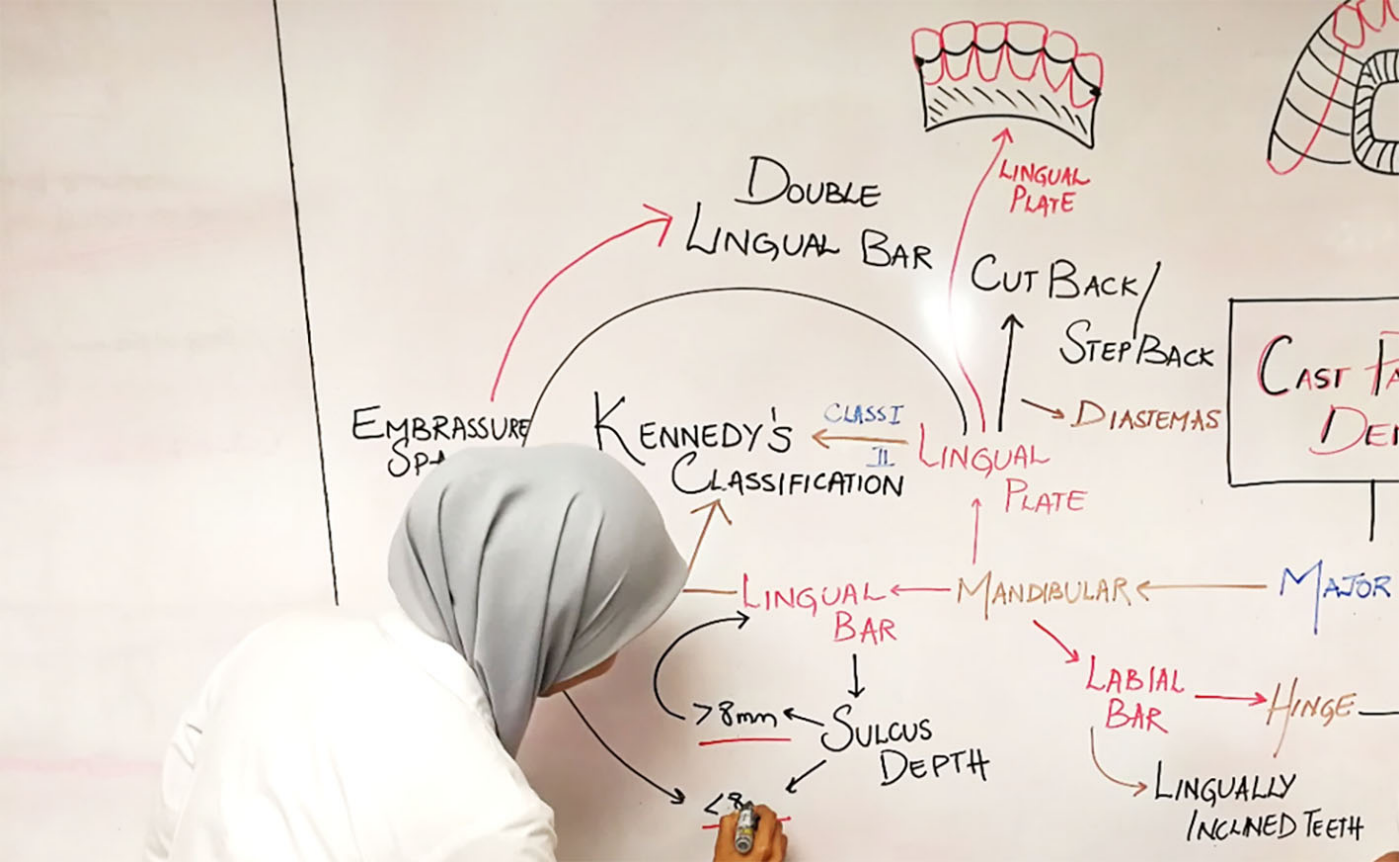
A student completing a Concept Map with missing nodes

**Figure 9.**
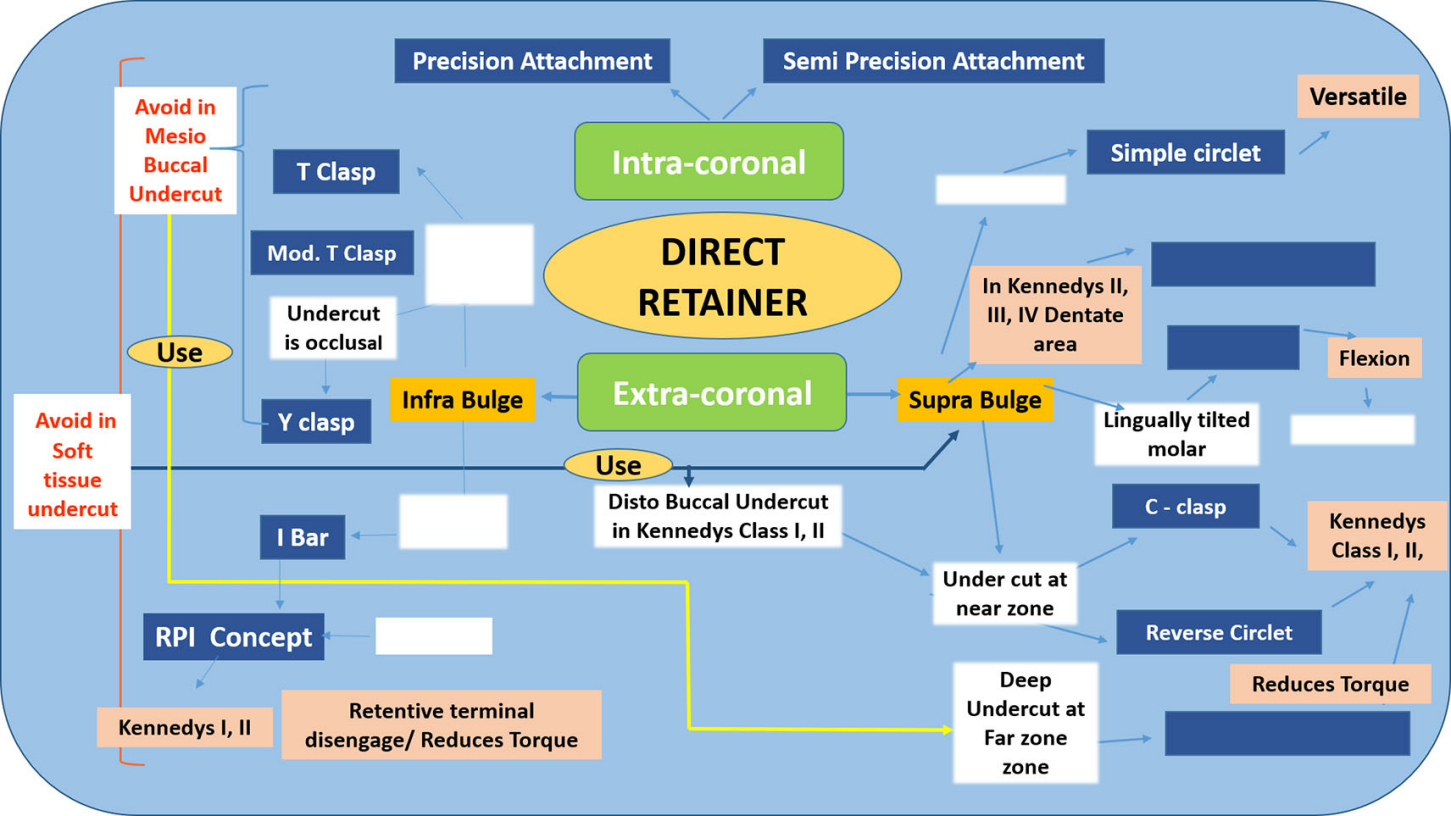
Concept Map with missing nodes and links

Additionally,

a. We created a map with empty nodes and students were asked to fill *in the content of the nodes* ( Figure 8).

b. We created a map where the students had to *fill in the linking words*

c. A mixed format was also used where we created a skeleton of the map and we urged the students to *fill in the nodes and linking words* ( Figure 9).

Initially students found it challenging to add linking words onto the pre-drawn concept map. This was because of their uncertain grasp of the relationship between the concepts. We made the students practice drawing concept maps with emphasis on using meaningful linking words and identifying cross-links. At the end of a four-week training session, spanning diverse topics, students were able to appreciate that every concept could be related to every other concept.

### Concept Maps for testing

Higher levels of cognitive performance can be achieved if concept mapping is used as an instrument of evaluation. It can become a powerful weapon in the assessment armamentarium, provided it is used sagaciously.

In the transition phase from a fledgling to a full-fledged physician, aberrations in the knowledge framework cannot be picked up by conventional examinations. However, concept maps are powerful in detecting these deviations and lacunae in students’ understanding ( West *et al*., 2002). Moreover, in the problem-based learning scenario, concept maps are effective tools to probe the knowledge structure ( Rendas, Fonseca and Pinto, 2006; Kassab and Hussain, 2010).

The scoring system for concept maps needs further study to address validity and reliability to use it as an assessment tool ( West *et al*., 2002; Ruiz‐Primo *et al*., 2004; Nesbit and Adesope, 2006; Ingeç, 2009). We followed the structural method for scoring the maps. All the participating students were trained in drawing concept maps. Then students were asked to draw a concept map as a part of assessment for a selected topic in Prosthodontics.

We modified the criteria given by Croasdell, Freeman and Urbaczewski (2003) and scored the maps using four categories, with the following point assignments for each valid component:

• Nodes representing the concepts (5 points each)
• Concept-link (2 points each)
• Cross-links (10 points each)
• Propositions (5 points each)

Three faculty individually rated the maps. A total structural score was obtained from the sum of scores from each component. A standard structural score was calculated by sum of structural scores given by the three faculty members. After the examination, a debriefing session was conducted to provide constructive feedback to students.

Concept maps drawn by some students clearly reflected their thinking. Some others became clearer when they were asked to explain orally. If not anything else, concept maps gave an opportunity for the students to talk about the topic conceptually, hopefully, in the process, enhancing their confidence and their understanding of the topic. This was a labor-intensive effort on the part of the faculty, but the rewards came in the opportunity it provided to gauge students’ understanding and plan appropriate pedagogical interventions.

### Concept Maps for thinking

Concept maps can be an impetus to the process of thinking. While applying the principles of problem solving, it is essential to make students visualize knowledge and think discerningly. This will facilitate sequential build-up while elevating the thinking to application level ( Latif *et al*., 2016).

**Figure 10.**
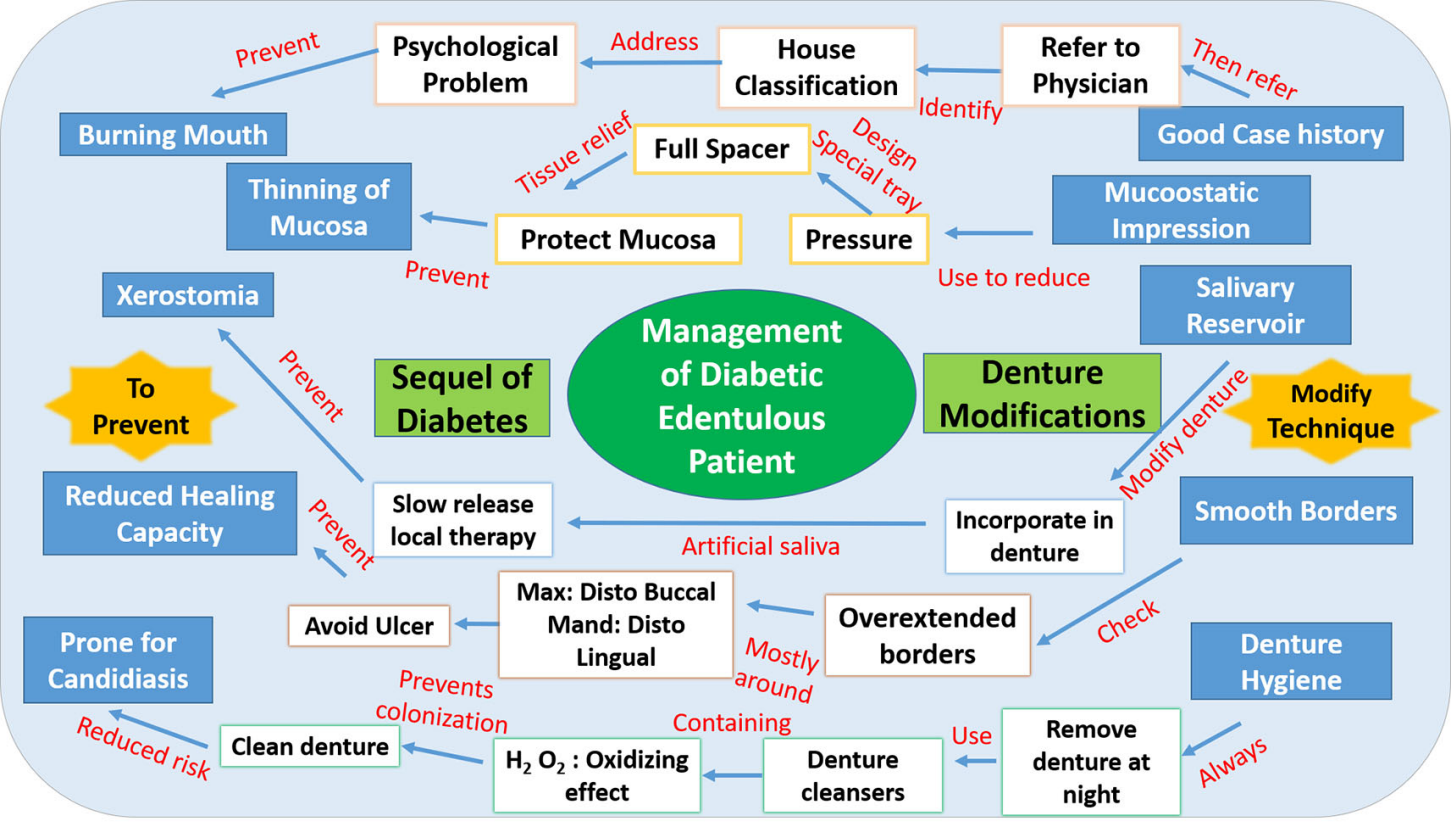
Example of a Concept Map drawn by the student

We chose concept mapping as a method to teach critical thinking in prosthodontics by selecting a case scenario (diabetic patient and impression making) and asking the students to integrate the two different concepts by linking them ( Figure 10).

We made the students do this exercise as a group task so that each member of the group contributed to the construction of the map. This exercise helped students understand the special considerations in impression making for a diabetic patient as it required logical reasoning and critical thinking during its construction.

## Conclusion

Usage of concept maps for teaching, training, testing and thinking impels students to construct a concept chain - with links functioning through relationships, yet each link maintaining its identity- this concept chain epitomizing a whole that mirrors the framework of concept thinking ( Reigeluth and Jonassen, 1999). Our educational voyage using concept maps for all these four purposes was not without impediments. However, we took comfort in the conviction that the journey is as important or perhaps more significant than the destination! Yes, students did grapple with ideas to identify meaningful cross links between concepts; this provided a platform for misconceptions to erupt which were revelations (sometimes startling!) for the teachers. Nevertheless, it was gratifying to see students making valiant but sincere attempts to link the concepts they were learning thus echoing the spirit of the first stanza in the poem by Arthur Hugh Clough: ( Tearle, 2020)

“Say not the struggle nought availeth,

The labour and the wounds are vain,

The enemy faints not, nor faileth,

And as things have been, they remain”.

## Take Home Messages

• Concept maps can be used for teaching, training, testing and thinking purposes.
• While using concept maps, conceptual reinforcement takes precedence over content coverage.
• Training students to represent their thinking in their concept maps is vitally important.
• Concept map is a powerful weapon in the assessment armamentarium, provided it is used sagaciously.
• Concept mapping allows sequential build-up of knowledge, while elevating the thinking to application level.

## Notes On Contributors

**Dr Prashanti Eachempati** is working currently as Professor and Head of Prosthodontics at Faculty of Dentistry, Melaka Manipal Medical College, Malaysia. She is a FAIMER fellow (GSMC India), and the recipient of the prestigious Ron Harden Innovation in Medical Education Award in 2017. She has been a keynote speaker at various national and international conferences, continuing professional development programmes and conducted workshops at various institutions in medical/dental education related topics. ORCID iD: https://orcid.org/0000-0003-1263-7423

**Dr Komattil Ramnarayan** was the fifth Vice-Chancellor of Manipal University (now known as Manipal Academy of Higher Education) from 2010 to 2015. He is currently the Chancellor of Manipal University Jaipur and Professor of Pathology at Melaka Manipal Medical College, Manipal Campus, India. He is also the National Coordinator (Pathology) for the National Program on Technology Enhanced Learning (NPTEL), a Government of India project. He was one of the early recipients of the ECFMG Foreign Faculty Fellowship in Basic Sciences. He was the recipient of the Bloomberg UTV Award for Outstanding Contribution to education. He has conducted more than 500 faculty development workshops in India and its neighboring countries. ORCID iD: https://orcid.org/0000-0002-7117-1830

**Dr Kiran Kumar KS** is working currently as an Professor in the Department of Prosthodontics at Faculty of Dentistry, Melaka Manipal Medical College, Malaysia. He has rich research experience, conducted several research projects, and has several Cochrane publications to his credit. He is involved in curriculum development, teaching and assessment of undergraduate dental students’ clinical skills, and reflective writing skills. ORCID iD: https://orcid.org/0000-0001-6638-0120

**Dr Anoop Mayya** is working currently as an Assistant Professor in the Department of Prosthodontics at Faculty of Dentistry, Melaka Manipal Medical College, Malaysia. He strives to make the classroom a space for growth, collaboration and inclusion and is actively involved in research, curriculum development, teaching, and assessment of undergraduate dental students’ clinical skills. ORCID iD: https://orcid.org/0000-0001-6583-4311
