# Supplementary material for: Concept Maps for Teaching, Training, Testing and Thinking
Source: MedEdPublish (2016). 2020 Aug 19;9:171. [Version 1] doi: 10.15694/mep.2020.000171.1 (PMC10702656; doi:10.15694/mep.2020.000171.1)
Supplement: Peer review history

# Reviewer report: Reviewer response for version 1

**DOI:** 10.21956/mep.19953.r30305

**Published:** 2020-09-08

**Authors:** P Ravi Shankar

**Affiliations:** 1 American International Medical University

This review has been migrated. The reviewer awarded 4 stars out of 5 This article starts by providing basic concepts about concept maps and then explores the use of concept maps for teaching, training, testing and thinking. The article provides basic information about concept maps in a simple and readable form supplemented by diagrams. The authors experience with concept maps is also of interest. Greater details about the use of concept maps at the author’s institution will be of interest. Further details about the use of the concept maps shown in Figures 7, 8, 9 and 10 will be of interest.

Reviewer Expertise:

NA

No decision status is available

# Reviewer report: Reviewer response for version 1

**DOI:** 10.21956/mep.19953.r30306

**Published:** 2020-08-28

**Authors:** Monika Sharma

**Affiliations:** 1 CMC Ludhiana , India

This review has been migrated. The reviewer awarded 3 stars out of 5 Concept maps is a great concept. Many of us would remember using something like a concept map when we tried to memorize complicated patho-physiological concepts. A better understanding of how a concept map is created would enhance learning for students at the undergraduate level. It is worth writing about it and introducing it to students. I read this article with interest and enjoyed the authors assimilation of available information about the concepts and utility of concept map.It was interesting to note their attempt of training their students to utilize concept maps. However, as this is presented as a 'case study', I was looking for more details of the 'case'. The article reads out more like a review of literature with a brief mention of what the authors did at their institute. I would like to read more about how they trained their faculty, not assuming that every one knows that perfectly. How did the faculty receive the concept of introducing concept mapping to the undergraduate students and how did the students perceive the concept? It goes without saying that a new concept impacts learning positively, hence it is too early to comment on how using concept maps as a tool for learning has been useful. Rather, I would like to read more about faculty and student feedback, if taken. The authors can consider writing a shorter introduction followed by presenting their work with concept maps and then discussing it with the available literature.

Reviewer Expertise:

NA

No decision status is available

# Reviewer report: Reviewer response for version 1

**DOI:** 10.21956/mep.19953.r30304

**Published:** 2020-08-24

**Authors:** Nuno Neuparth

**Affiliations:** 1 Nova Medical School

This review has been migrated. The reviewer awarded 3 stars out of 5 Writing about concept mapping is slowly becoming a more frequent topic in health education. For that reason, all the information made public about the use and experience with concept mapping, reporting undescribed experiences and supporting new approaches, is worthwhile. Recently, our group presented in MedEdPublish (2) a proposal of a template to be used in concept mapping in medical education as part of our ongoing research project in this area which was even more very recently complemented by another publication of different depth and breadth (1). In the present case, starting with a description of the use of concept mapping based on examples and figures is relevant for the local team but it needs to be reinforced by broad research questions, particularly in the area of evaluation in order to be useful for the health education community. As Pudelko et al (3) clearly state: “Current empirical research on mapping as a learning strategy presents methodological shortcomings that limit internal and external validity”. In this revived and fascinating field of concept mapping, with no single solutions and multiple approaches, we are all beginners and need to humbly learn as much as we can from each other.Bibliografia1. Marta Fonseca, Beatriz Oliveira, Pedro Carreiro-Martins, Neuparth N, and Rendas aA. Revisiting the role of concept mapping in teaching and learning pathophysiology for medical students Adv Physiol Educ 44: 475-481, 2020.2. Marta Fonseca BO, Pedro Carreiro-Martins, Nuno Neuparth, Antonio Rendas. A concept map template to be used by medical students for displaying pathophysiological mechanisms within clinical cases MedEdPublish 9: 2020.3. Pudelko B, Young M, Vincent-Lamarre P, and Charlin B. Mapping as a learning strategy in health professions education: a critical analysis. Med Educ 46: 1215-1225, 2012.

Reviewer Expertise:

NA

No decision status is available

# Reviewer report: Reviewer response for version 1

**DOI:** 10.21956/mep.19953.r30308

**Published:** 2020-08-23

**Authors:** Megan Anakin

**Affiliations:** 1 University of Otago

This review has been migrated. The reviewer awarded 3 stars out of 5 I read this case study with interest because I am familiar with using concept maps as a learning tool. I appreciated reading about your experiences and recommendations about using concept maps. Health professional educators will appreciate reading the descriptions of how you used concept maps with your students.I have a few suggestions that might help to enhance your article. To help the reader appreciate the first sentence in the introduction, please consider identifying which “cognitive processes undergirding teaching- learning” and explain how they “are so distinctly dissimilar” to support your claim “that it is important to use variegated tools to enhance knowledge construction in learners”. Please consider identifying a few pedagogical changes that have been propounded and describe their strengths and limitations related to the cognitive processes they emphasise so that the reader understands why this article is focused on concept maps as an appropriate pedagogical intervention. To strengthen the section about the initiation of concepts maps, please consider using direct references Ausubel’s work to support your claims about his “meaningful learning theory”. In the section titled ‘Our experience with concept maps’, please explain the features of the evidence in the literature that supports concept mapping “as a meta-cognitive strategy that enhances meaningful learning”. Also in this section, there is a two-paragraph description of an experimental study conducted by the authors about the impact of concept mapping on student reasoning. Please consider describing the methods and results in greater detail so the reader can better assess the meaningfulness of the claims made about the experimental study’s results. To help the reader appreciate and be able to use the scoring criteria presented in the testing section of the article, please explain how a concept map would earn points in each of the four categories and why it was necessary to sum the scores from three assessors. In the conclusion, the authors note that there were impediments. Please consider discussing these impediments in a discussion section before the concluding paragraph. In the take home messages, please consider revising each point to better represent the evidence from practice presented in this article. For example, please provide a brief reason why training students to represent their thinking in their concept maps is vitally important.To enhance the connection between the title and the beginning of the article, please consider signposting and defining the four functions of concept maps (for Teaching, Training, Testing and Thinking). Please explain also how these four functions link to cognitive processes in the introduction section of the article to prepare the reader for the examples from practice in the later sections of the article.I would be very happy to review a revised version of this article and I would like to encourage you to submit one.

Reviewer Expertise:

NA

No decision status is available

# Reviewer report: Reviewer response for version 1

**DOI:** 10.21956/mep.19953.r30307

**Published:** 2020-08-19

**Authors:** Richard J. Nierenberg

**Affiliations:** 1 Hackensack University Medical Center, Hackensack Meridian Health

This review has been migrated. The reviewer awarded 4 stars out of 5 This is an interesting introduction and summary of the notion of concept mapping to educate students in determining and organizing information to develop expertise.There is a somewhat lengthy introduction of the concept leading to several linked proprietary websites which are instructive. This introduction could have been more streamlined. The initial chart, which includes concepts along with authors in the place of concepts does not further the concept as well as subsequent charts.The strength of the article seems to be in its demonstration of the nuts and bolts of a concept map. This is introduced with a black board image of the analysis of hypertension, which is stimulating but hard to assess. The more comprehensive chart is, however, done with the orthodontic literature, which limits its application somewhat.I believe if the authors took either one of those charts and in narrative form "walked us through" how the use of the concept map would broaden understanding for a student and therefore aspiring expert would make the article even more useful.Still it is a comprehensive, and enticing introduction and presentation of an interesting and engaging pedagogical process, and worth the read.

Reviewer Expertise:

NA

No decision status is available
